# Altered Eye-Movement Patterns During Text Reading in Obsessive–Compulsive Disorder and Internet Gaming Disorder

**DOI:** 10.3389/fnbeh.2018.00248

**Published:** 2018-10-18

**Authors:** Tak Hyung Lee, Minah Kim, Yoo Bin Kwak, Wu Jeong Hwang, Taekwan Kim, Jung-Seok Choi, Jun Soo Kwon

**Affiliations:** ^1^Department of Brain and Cognitive Sciences, Seoul National University College of Natural Sciences, Seoul, South Korea; ^2^Department of Psychiatry, Seoul National University College of Medicine, Seoul, South Korea; ^3^Department of Psychiatry, SMG-SNU Boramae Medical Center, Seoul, South Korea; ^4^Institute of Human Behavioral Medicine, SNU-MRC, Seoul, South Korea

**Keywords:** information processing, eye-movement, reading, obsessive-compulsive disorder, internet gaming disorder

## Abstract

Obsessive-compulsive disorder (OCD) and internet gaming disorder (IGD), which are similar in that both involve repetitive behaviors and related with cognitive dysfunctions, frequently begin in early adolescence, which is a critical period for learning. Although the deterioration in cognitive functioning caused by these conditions may have adverse effects on information processing, such as text reading, there has been no comprehensive research on the objective indicators of altered reading patterns in these patients. Therefore, we evaluated eye-movement patterns during text reading in patients with OCD or IGD. In total, 20 patients with OCD, 28 patients with IGD and 24 healthy controls (HCs) participated in the reading task using an eye tracker. We compared the fixation durations (FDs), saccade amplitudes and eye-movement regressions of the three groups during reading. We explored relationships between the parameters reflecting altered reading patterns and those reflecting the severity of clinical symptoms. The average FDs and forward saccade amplitudes did not differ significantly among the groups. There were more eye-movement regressions in patients with OCD than in patients with IGD and HCs. No correlation was found between altered eye-movement patterns during reading and the severity of clinical symptoms in any of the patient groups. The significantly increased number of regressions (NRs) in the OCD group during reading may reflect these patients’ difficulties with inferential information processing, whereas the reading pattern in the IGD group is relatively intact. These findings suggest that patients with OCD and patients with IGD have different eye-movement patterns during reading reflecting distinct cognitive impairments in the two patient groups.

## Introduction

Obsessive-compulsive disorder (OCD) and internet gaming disorder (IGD), the most prevalent conditions among adolescents, share symptoms involving repetitive behavior (Walitza et al., 2011; Robbins and Clark, 2015; Feng et al., 2017). OCD is characterized by obsessions and compulsions, which are intrusive, recurring, unwanted, or aversive thoughts and repetitive, ritualized behavior or mental activity that is aimed at reducing distress (American Psychiatric Association and American Psychiatric Association. DSM-5 Task Force, 2013). IGD, which is characterized by a lack of interest in all everyday activities except internet games, was categorized as a condition requiring further study in Section III of the Diagnostic and Statistical Manual of Mental Disorders-5 (DSM-5) in 2013 (American Psychiatric Association and American Psychiatric Association. DSM-5 Task Force, 2013; King and Delfabbro, 2014). These two conditions are similar in that both involve repetitive thoughts and behaviors and cognitive dysfunction, including impaired inhibitory control (Walitza et al., 2011; de Wit et al., 2012; King and Delfabbro, 2014) and the failure to resist an impulse or temptation to perform an act that is harmful to the person (Grant et al., 2010). From this perspective, OCD and IGD can be categorized as behavioral addictions (Holden, 2001; Potenza, 2006; Robbins and Clark, 2015). Patients with OCD have impairments in various domains of cognitive functioning, such as inhibitory control, set shifting, planning and working memory (Dittrich and Johansen, 2013; Shin et al., 2014; Abramovitch and Cooperman, 2015). Excessive internet use and difficulties in suppressing the cravings for internet games are associated with executive dysfunction in patients with IGD (Dong and Potenza, 2014; Zhou et al., 2016). Impaired visuospatial memory (VSM) is the most consistently reported cognitive impairment in patients with OCD. However, VSM deficits are rarely reported in patients with IGD. Instead, VSM may be enhanced by the repetitive exposure to visual stimuli and cognitive training involved in internet gaming (Oei and Patterson, 2013; Blacker et al., 2014).

Both OCD and IGD are most prevalent during early adolescence, which is a critical period for learning and social development (Kessler et al., 2007). In addition, both OCD and IGD has repetitive thought and attentional bias to a specific object or target related with their symptom (Tata et al., 1996; Bradley et al., 2016; Kim et al., 2018). Because those symptoms affect attentional capacity which is a very basic unit of cognitive functioning, broad range of cognitive functioning can be affected by symptoms of OCD and IGD. This is particularly important because the cognitive impairments that affect information processing could have a negative impact on normal development (Millan et al., 2012). Indeed, impaired cognitive functions in early adolescence could affect individuals in achieving educational success; this may in turn lead to later social and occupational dysfunction, which may reduce the long-term quality of life (Sawyer et al., 2002). One of the most important learning-related abilities in school-age children involves reading, which is affected in both OCD and IGD by impairments in higher order cognitive functioning (Carretti et al., 2009; Borella et al., 2010). Reading skills are essential for learning and good academic performance, as a large proportion of new information is acquired through reading (Pretorius, 2002; García-Madruga et al., 2014). Reading requires the comprehensive use of various domains of cognitive functioning, such as working memory, inhibitory control, lexical processing and attentional control. Therefore, reading problems may develop in both patients with OCD and those with IGD because of the aforementioned impairments in information processing or cognitive functioning.

The eye-movement patterns involved in the information processing underpinning the process of reading were recently measured using an eye tracker (Raney et al., 2014; Cop et al., 2015). The basic assumption of such a measurement method is that longer (i.e., the amount of time that a gaze is fixed on a certain position) and more numerous fixations are associated with longer periods of information processing (Raney et al., 2014). Excellent readers have short fixation durations (FDs), long saccades (i.e., gaze movements between fixations) and few instances of repetitive reading (regressions; Rayner, 2009). A study by Deans et al. (2010) found that patients with reading disorders (RDs) spent a longer total time reading and had longer FDs than healthy controls (HCs); additionally, patients with attention-deficit/hyperactivity disorder (ADHD) had a higher proportion of regressive and vertical saccades compared to HCs (Deans et al., 2010). Another study on RDs identified regressive saccades as a factor that could differentiate individuals with RDs from HCs (Nilsson Benfatto et al., 2016). Finally, in one study there were more regressions and more and longer fixations among those in the early stages of Alzheimer’s disease compared to HCs (Fernández et al., 2013). Thus, the eye movements performed during reading may reflect changes in the cognitive processes involved in text comprehension associated with various diseases characterized by cognitive impairments.

OCD and IGD are both marked by dysfunctional information processing because of impaired cognitive functions. However, the eye-movement patterns during reading may differ in individuals with these conditions because of the unique characteristics of each. VSM is known to be impaired in OCD (Shin et al., 2004; Abramovitch et al., 2013) but not in IGD, as a previous study reported that VSM may be enhanced by the repetitive exposure to visual stimuli and cognitive training involved in internet gaming (Blacker et al., 2014; Steenbergen et al., 2015). Indeed, patients with OCD may be more likely to repeatedly read the same text because of comprehension difficulties caused by either obsessions or compulsions that render set shifting difficult or create a slow processing speed (Abramovitch et al., 2013). In contrast, patients with IGD may have faster reading speeds despite comprehension difficulties due to their repeated exposure to various images and game scenes during game play.

Therefore, we used an eye-tracking method to investigate whether the eye-movement patterns during text reading differed between groups with OCD and IGD. We hypothesized that, during reading, patients with OCD would have longer FDs and more numerous regressions than patients with IGD, whereas the reading patterns of patients with IGD would be relatively preserved despite their dysfunctional information processing.

## Materials and Methods

### Participants and Clinical Assessments

Twenty patients with OCD, 28 patients with IGD, and 24 HCs participated in this study. Patients with OCD were recruited from the outpatient clinic at Seoul National University Hospital (SNUH). Patients with IGD were recruited from the addiction outpatient clinic at SMG-SNU Boramae Medical Center. OCD and IGD were diagnosed by experienced psychiatrists based on *DSM-5* criteria. Eight patients with OCD were taking selective serotonin reuptake inhibitors (SSRIs) at the time of the study. We evaluated the severity of obsessive-compulsive (OC) symptoms using the Yale-Brown Obsessive Compulsive Scale (Y-BOCS; Goodman et al., 1989). All patients with IGD played internet games for more than 4 h per day for 1 year and were medication free. HCs played internet games for no more than 2 h per day and had no history of psychiatric illness. The severity of all participants’ internet gaming addiction was assessed using Young’s Internet Addiction Test (IAT; Young, 1996). The Barratt Impulsivity Scale-11 (BIS-11; Fossati et al., 2001) was used to measure impulsivity. We assessed the severity of depressive symptoms using the Beck Depression Inventory (BDI; Steer et al., 1999), and the severity of anxiety symptoms was assessed using the Beck Anxiety Inventory (BAI; Steer et al., 1993). We evaluated the intelligence quotient (IQ) using the abbreviated form of the Korean-Wechsler Adult Intelligence Scale-III (K-WAIS-III; Kim et al., 1994). Exclusion criteria included lifetime diagnosis of substance abuse or dependence, neurological disease, significant head injury accompanied by loss of consciousness, medical illness with documented cognitive sequelae, sensory impairment, or intellectual disability (IQ <70).

This study was carried out in accordance with the recommendations of GCP, the institutional review boards of SMG-SNU Boramae Medical Center and SNUH. The protocol was approved by the the institutional review boards of SMG-SNU Boramae Medical Center and SNUH. All subjects gave written informed consent in accordance with the Declaration of Helsinki.

### Reading Task and Eye-Movement Recording

The content of the reading task was selected from among the reading assignments provided by the Educational Broadcasting System of Korea (EBS). To ensure that the assignment would be easily understood by both adolescent and adult participants, we chose a natural science text that had elicited a rate of response to comprehension questions of more than 80% correct. This text addressed the mechanism of heavy rain formation, and the reading task was developed using Experimental Builder 2.1.45 (SR Research, Canada). Reading assignments were displayed on a 17 inches monitor with dimensions of 1024 × 768 pixels. The reading task consisted of five pages with 10 lines per page, yielding 44 lines in total. After finishing reading one page, subjects proceeded to the next page by clicking the mouse button. Eye movements were measured using EyeLink 1,000 (SR Research), with a 1,000 Hz sampling rate, while participants performed the reading task. The font size and line spacing were about 1°, the horizontal viewing angle was 27°, and the vertical viewing angle was 20°. Participants placed their jaws and foreheads on a chin rest at a distance of 70 cm from the monitor. To ensure that participants performed the task to the best of their ability, before starting the experiment we told them that they would be asked four questions related to the reading after finishing the task.

### Eye-Movement Data Analyses

**Figure 1:**
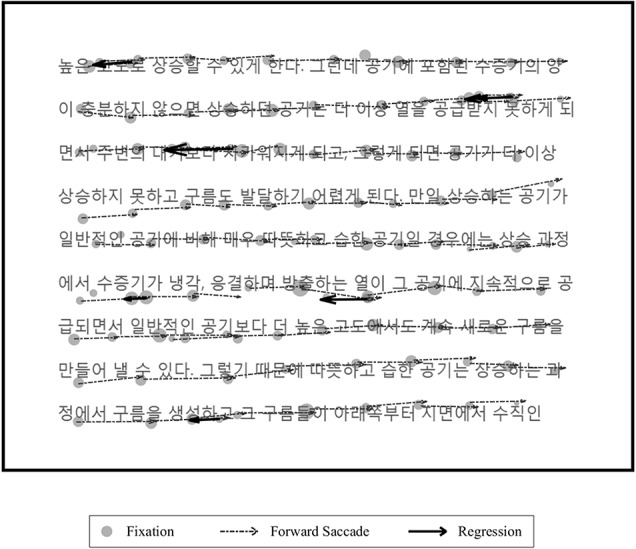
Image of the sample scene of the reading task with the eye-movement variables superimposed. The text, written in Korean in the box in bold, was presented in the task. Gray circles mean that the fixation and size of the circle varied according to the duration of each fixation. Arrows with dotted lines indicate forward saccades. Arrows with bold lines indicate regressions.

Data analyses were performed using EyeLink Data Viewer 2.6.1 (SR Research) and customized MATLAB scripts. The variables of interest were FD, amplitude of forward saccades (AFS) and number of regressions (NRs). Fixations and saccades were identified by EyeLink 1,000 tracker parser processes. The parser processes differed from the manufacturer’s settings, which were calibrated to the threshold values for displacement, velocity, and acceleration, which were set at 0.15°, 30°/s and 8,000°/s^2^, respectively. FD is the value of the duration of fixed gazes. FSA is the size of the angle formed by the movement of the eye toward the text. NR reflects the number of times that the saccade traveled in the direction opposite the direction of text progression. Figure 1 presents an example of the fixations, saccades, and NR superimposed over a screen displaying the reading task.

### Statistical Analyses

Data were analyzed using the lmerTest and multcomp packages of R. We used one-way analysis of variance (ANOVA) to compare demographic and clinical characteristics and some of the eye-movement parameters (i.e., total duration and NR) across the three groups. Because FD and AFS had a skewed long-tail distribution in individuals, we used the median value for each subject for the Kruskal-Wallis test. We analyzed categorical data using chi-square tests. *Post hoc* Bonferroni tests were performed if indicated. Pearson’s correlation analyses were performed to explore associations between altered eye-movement parameters during reading and scores on the Y-BOCS or IAT of each patient group. Also, to rule out the effect of depressive symptoms, we performed an exploratory correlation analysis between BDI scores and NR, both of which were significantly larger in OCD patients than the IGD group and HCs. The level of statistical significance was set at *P* < 0.05.

## Results

### Demographic and Clinical Characteristics

There were no significant differences among the groups in terms of sex, age, years of education, IQ, or BIS-11 score. However, there were significant intergroup differences in IAT (*F* = 16.481, *P* < 0.001), BDI (*F* = 13.373, *P* < 0.001), and BAI (*F* = 10.093, *P* < 0.001) scores. *Post hoc* Bonferroni testing revealed that patients with IGD (*t* = 5.637, *P* < 0.001) and those with OCD (*t* = 3.045, *P* = 0.010) had higher IAT scores than HCs. The BDI score was highest in the OCD group (OCD vs. IGD, *t* = 2.638, *P* = 0.031; OCD vs. HC, *t* = 4.904, *P* < 0.001), intermediate in the IGD group (IGD vs. HCs, *t* = 2.632, *P* = 0.032), and lowest in HCs. The BAI score was higher in the IGD (*t* = 3.430, *P* = 0.003) and OCD (*t* = 4.057, *P* < 0.001) groups compared to HCs (Table 1).

**Table 1:** Demographic, clinical characteristics of participants.

				Statistical Analysis^a^	
	OCD (*n* = 20)	IGD (*n* = 28)	HC (*n* = 24)	*F* or *χ*^2^	*P*
Demographics					
Sex (Male/Female)	16/4	27/1	18/6	5.062	0.080
Age (years)	25.4 (5.6)	26.3 (5.1)	25.1 (4.9)	0.378	0.686
Education (years)	13.8 (1.3)	14.2 (1.9)	14.3 (1.4)	0.623	0.539
IQ	104.3 (27.8)	107.4 (15.3)	116.0 (8.1)	2.553	0.085
Clinical characteristics					
IAT	45.4 (19.3)	54.8 (11.9)	31.6 (8.8)	16.481	<0.001***
BDI^b^	15.8 (10.7)	9.5 (6.5)	4.2 (4.3)	13.373	<0.001***
BAI^b^	12.3 (8.9)	10.0 (7.8)	3.2 (3.6)	10.093	<0.001***
BIS-11^b^	64.8 (9.0)	62.4 (8.7)	58.7 (6.9)	3.12	0.051
Y-BOCS total	23.8 (6.8)	NA	NA	NA	NA
Obsession	11.9 (3.6)	NA	NA	NA	NA
Compulsion	11.9 (3.7)	NA	NA	NA	NA

Data are given as mean (standard deviation). ***P < 0.001. Abbreviations: OCD, obsessive-compulsive disorder; IGD, internet gaming disorder; HC, healthy control; IQ, intelligent quotient; IAT, Korean version of the Young internet addiction test; BDI, Beck depression inventory; BAI, Beck anxiety inventory; BIS-11, Barratt impulsiveness scale version 11; Y-BOCS, Yale-Brown obsessive-compulsive scale; NA, Not Applicable. ^a^Analysis of variance or χ^2^ analysis. ^b^With one missing values in OCD group.

### Reading Task and Eye Movements

**Figure 2:**
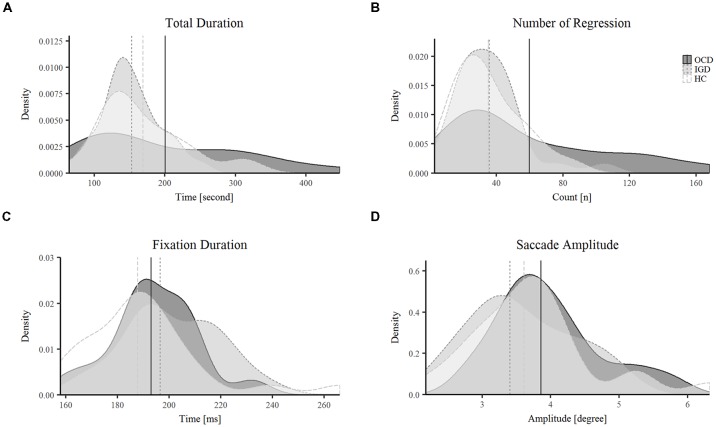
The distribution of data by group for total duration **(A)**, number of regression (NR) **(B)**, fixation duration (FD) **(C)** and saccade amplitude **(D)**. In each plot, solid line indicates the OCD group and dotted line indicates the IGD group and the dashed line indicates the HC group. The vertical line indicates the mean value of the group in **(A)** and **(B)**. In **(C)** and **(D)**, the vertical line indicates the median value of each groups Abbreviations: OCD, Obsessive-compulsive disorder; IGD, internet gaming disorder; HC, healthy control.

**Figure 3:**
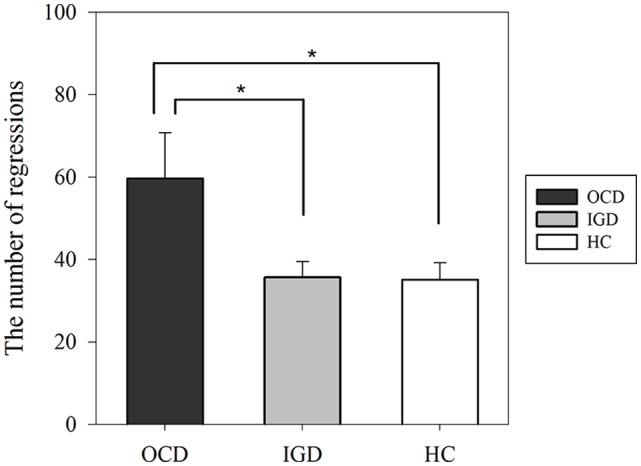
NRs in the three groups according to *post hoc* analyses using the Bonferroni method. The error bar indicates the standard error. *P < 0.05. Abbreviations: OCD, obsessive-compulsive disorder; IGD, internet gaming disorder; HC, healthy control.

The eye-tracking data of two patients with OCD who correctly answered only one of the four questions related to the reading were excluded from further analyses because the seriousness of their participation seemed questionable. The means (standard deviation) of skewness of all subjects were 1.34 (0.99) for FD and 2.66 (1.14) for the AFS. There were no significant intergroup differences in the total duration of reading (*F* = 2.479, *P* = 0.091), FD (χ^2^ = 5.748, *P* = 0.056), or AFS (χ^2^ = 2.591, *P* = 0.274). However, we found significant group differences in NR (*F* = 4.553, *P* = 0.014; Table 2). The distribution of data by group for the four variables is shown in Figure 2. *Post hoc* Bonferroni testing revealed that patients with OCD had higher NR than patients with IGD (*t* = 2.702, *P* = 0.026) and HCs (*t* = 2.678, *P* = 0.028) as shown in Figure 3. There were no significant correlations between NR and Y-BOCS total score (*r* = 0.203, *P* = 0.362) or between scores on the BDI and NR (*r* = −0.016, *P* = 0.948) in patients with OCD. Additionally, we performed analysis of covariance (ANCOVA) to the total duration and the NR, because the IQ showed the trend level group difference. In the result of ANCOVA, group differences in NR remained significant after IQ controlled as a covariate (*F* = 4.504, *p* = 0.015).

**Table 2:** Reading task performing duration and eye-movement analysis results during reading task across three groups.

				Statistical Analysis^a^	
	HC (*n* = 24)	IGD (*n* = 28)	OCD (*n* = 18)	*F* or *χ*^2^	*P*
Total duration^a^(s)	168.8 (62.0)	153.0 (40.7)	200.5 (109.4)	2.479	0.091
Number of regression^a^ (*n*)	35.1 (20.0)	35.7 (20.5)	59.7 (47.0)	4.553	0.014*
Fixation duration^b^ (ms)	187.8 (175.2; 199.0)	196.5 (190.5; 215.0)	193 (187.0; 206.5)	5.748	0.056
Saccade amplitude^b^ (ms)	3.6 (3.2; 4.0)	3.4 (3.1; 4.2)	3.9 (3.4; 4.3)	2.591	0.274

Abbreviations: HC, healthy control; IGD, internet gaming disorder; OCD, obssesive-compulsive disorder. ^a^Analysis of variance. Data are given as mean (standard deviation). *P < 0.05. ^b^Kruskal-Wallis rank sum test. Data are given as median (1 quantile; 3 quantile).

## Discussion

We aimed to identify the distinctive patterns of eye movements of patients with OCD and patients with IGD during reading. We found that the time spent on lexical processing (i.e., FD) and the distance traveled when the gaze moved according to the direction of the text (i.e., AFS) did not differ significantly among the groups. However, the number of returns from the direction of reading (i.e., NR) was significantly higher in patients with OCD than in patients with IGD and HCs. There was no significant correlation between NR and the severity of OC symptoms in patients with OCD.

A more significant increase in NR was found in the OCD group compared to the IGD and HC groups. Regressions occur when readers miss, forget, or are unsure about what they have already read, and NR is influenced by the difficulty of the text (Rayner, 1998; Booth and Weger, 2013). Mathematical eye-movement models, such as saccade-generation with inhibition by foveal targets (SWIFT) and E–Z reader, also indicate that regression occurs when it is difficult to understand a specific word in a sentence (Engbert et al., 2002; Reichle et al., 2003). The increased NR during reading shown by patients with OCD suggests that their cognitive dysfunction may have impaired their ability to understand the content of text that they had read before moving their gaze (Cop et al., 2015). During text reading, most information processing occurs during fixations (Rayner, 2009). Only the OCD group showed increased NR in the current study, and the trend level of the FD of the three groups differed; thus, the ability of the OCD group to process information differed with that of other groups. Additionally, the reduced inhibitory control and set shifting ability related to the repetitive behavioral characteristics of OCD may have contributed to the increased NR in the OCD group; that is, the habitual forward movement of the gaze of patients with OCD, even when they had gained sufficient understanding of the sentence they had just read, may have affected these results. Moreover, patients with OCD may have reconfirmed their understanding of text they had already read because of a deterioration in their confidence in their cognitive abilities (Hermans et al., 2008). However, the lack of increased NR in patients with IGD may reflect the stability of the confidence of these patients and the fact that the only repetitive activities performed by this group involve internet games.

In contrast to our initial hypothesis, we did not find a significant correlation between increased NR and the severity of OC symptoms in patients with OCD. This may have been because of the trait status of abnormal reading patterns, which would mean that such patterns would not change as a function of a state change, such as symptomatic improvement. Because dysfunctions in several cognitive domains are traits of OCD, reading ability, which is closely related to cognitive functioning, is also likely to be a trait marker rather than a state marker (Abramovitch and Cooperman, 2015). Another possible explanation involves the cognitive complexity of eye-movement patterns during reading. Reading requires integrating and regulating many domains of cognitive functioning, including working memory, attention, word identification and language comprehension (Fernández et al., 2014). Therefore, it may be difficult to identify correlations between simple eye-tracking parameters and symptom severity without considering the complex interactions of various domains of cognitive functioning that may occur during reading. The heterogeneity of the severity and characteristics of OC symptoms may also have contributed to the inconsistency between the results and the initial hypothesis. Although it is likely that NR is associated with repetitive behavior, some patients with OCD may suffer from obsessions in the absence of repetitive compulsive behaviors. In addition, many of the patients with OCD were taking medication, which may have reduced the severity of their symptoms to below the threshold of detection of our eye-tracking method. Such heterogeneity could have obscured the possible correlation between increased NR and the severity of OC symptoms.

In the current study, patients with IGD, unlike those with OCD, did not show altered eye-movement patterns related to impaired reading ability. IGD involves both compulsive characteristics (i.e., repetitive gaming behavior) and addictive characteristics (i.e., an increased desire to play a specific game; Dong and Potenza, 2014). The addictive characteristics may cause significant behavioral changes and cognitive abnormalities when a stimulus or desire related to the game of choice appears (Zhou et al., 2012); however, the domains of cognitive functioning that are impaired and/or the psychiatric symptoms that emerge may differ according to the characteristics of the game of choice (Na et al., 2017). For example, individuals addicted to first-person shooter (FPS) games have higher levels of impulsivity than nonaddicted persons (Metcalf and Pammer, 2014), and players of massive multiplayer online role-playing games (MMORPGs) have shown increased social anxiety (Park et al., 2016). However, playing video games improves visuospatial functions through repetitive training using visual cues (Oei and Patterson, 2013; Blacker et al., 2014), and regular playing of FPS games may improve decision-making ability or action cascading (Metcalf and Pammer, 2014; Steenbergen et al., 2015). MMORPGs may have positive effects on language learning through real-time online interactions with other players and the narrative or instructions embedded in the games (Zhang et al., 2017). Because the patients with IGD who participated in this study were addicted to games with different characteristics, the effects of addiction on eye-movement patterns during reading may have canceled themselves out, leading to an ostensible absence of abnormality. Another possible explanation is that because the patients with IGD in this study were recruited via internet advertisements, they may have had less severe addictions than participants in other studies, who were chosen from among those visiting a hospital for help and treatment (Kim et al., 2012; Zhou et al., 2016). Thus, the symptoms of the patients with IGD may not have been sufficiently severe to lead to measurable changes in reading patterns in the current study.

Because eye-movement patterns during reading reflect both the linguistic features of the text (i.e., word frequency, word length and sentence complexity) and the characteristics of the reader (i.e., reading ability and topic knowledge), they can be used to measure the text comprehension of a reader (Palmer et al., 1985; Rayner, 1998; Clifton et al., 2016). The altered eye-movement pattern of the OCD group implies an impaired reading ability that has also been observed in other psychiatric disorders, such as ADHD, RD and Alzheimer’s disease (Deans et al., 2010; Fernández et al., 2013). Moreover, these same abnormalities are reflected in the increased NR observed during reading. However, other features, such as the increased average fixation time in RDs and the total fixation and duration times, vary according to conditions (Fernández et al., 2013, 2014). Condition-related differences in eye-movement patterns during reading may be due to the different types and degrees of cognitive dysfunction associated with each disorder. Therefore, eye-movement tests, including those that measure the cognitive factors that may be affected by the pathological features of the condition under examination, are likely to be useful behavioral biomarkers in a variety of psychiatric patients (Itti, 2015).

This study has several limitations. First, we did not directly assess the reading comprehension of the participants, as the number of correct answers to the four questions was used to judge only the seriousness with which the task was performed. Therefore, this study did not address important aspects of information processing reflected in text comprehension. Second, the patient groups in this study were heterogeneous in terms of symptom severity, medication status and symptom domain (i.e., main OC symptom domain or game of choice). Specifically, the high BDI scores of the OCD group may have contributed to the increased NR even though there was no significant correlation between BDI scores and NR in OCD subjects. Eight patients with OCD were taking SSRIs, but their main symptom domains were heterogeneous. Patients with IGD played various types of games and had relatively low IAT scores. Such heterogeneity (including the use of SSRIs by patients with OCD (Sayyah et al., 2016)) and the relatively low level of symptom severity in patients with IGD may have affected participants’ information processing. Finally, the predominance of males in the three groups, especially the IGD group, and the relatively small sample size were other limitations of the current study.

To the best of our knowledge, this is the first study to differentiate altered eye-movement patterns of patients with OCD and patients with IGD according to characteristics of these diseases. We found that NR was increased in the OCD group, although there was no obvious change in the eye-movement pattern of the IGD group during text reading. The increased NR in the OCD group during reading may reflect difficulties with inference or set shifting due to characteristics of OCD. The relatively preserved eye-movement pattern of patients with IGD during text reading, despite their difficulties with information processing, may reflect the effects of cognitive—visual training during repeated game play. The findings of the current study suggest that patients with IGD and patients with OCD have different eye-movement patterns during reading that may reflect the distinct domains of cognitive dysfunction associated with each of these disorders. Additional studies that combine measurements of eye-movement patterns during reading with explorations of various domains of cognitive functioning would be of great interest.

## Author Contributions

MK and J-SC was responsible for recruitment of patients and HC participants, the collection of demographic and clinical data. TL, MK, J-SC and JK contributed for study design and procedure. TL, YK, WH and TK collected eye-movement data. TL and MK performed the data analysis and wrote the manuscript draft. J-SC and JK supported interpretation of the study results. J-SC and JSK managed and supervised the whole procedure of this study. All authors have critically reviewed the content and approved the final version of the manuscript.

## Conflict of Interest Statement

The authors declare that the research was conducted in the absence of any commercial or financial relationships that could be construed as a potential conflict of interest.

## References

[B1] Abramovitch A. Abramowitz J. S. Mittelman A. (2013). The neuropsychology of adult obsessive-compulsive disorder: a meta-analysis. Clin. Psychol. Rev. 33, 1163–1171. 10.1016/j.cpr.2013.09.004 24128603

[B2] Abramovitch A. Cooperman A. (2015). The cognitive neuropsychology of obsessive-compulsive disorder: a critical review. J. Obsessive. Compuls. Relat. Disord. 5, 24–36. 10.1016/j.jocrd.2015.01.002

[B3] American Psychiatric Association American Psychiatric Association. DSM-5 Task Force (2013). Diagnostic and Statistical Manual of Mental Disorders: DSM-5. 5th Edn. Arlington, VA: American Psychiatric Publishing, Inc.

[B5] Blacker K. J. Curby K. M. Klobusicky E. Chein J. M. (2014). Effects of action video game training on visual working memory. J. Exp. Psychol. Hum. Percept. Perform. 40, 1992–2004. 10.1037/a0037556 25068696

[B6] Booth R. W. Weger U. W. (2013). The function of regressions in reading: backward eye movements allow rereading. Mem. Cognit. 41, 82–97. 10.3758/s13421-012-0244-y 22886737

[B7] Borella E. Carretti B. Pelegrina S. (2010). The specific role of inhibition in reading comprehension in good and poor comprehenders. J. Learn. Disabil. 43, 541–552. 10.1177/0022219410371676 20606207

[B8] Bradley M. C. Hanna D. Wilson P. Scott G. Quinn P. Dyer K. F. W. (2016). Obsessive-compulsive symptoms and attentional bias: an eye-tracking methodology. J. Behav. Ther. Exp. Psychiatry 50, 303–308. 10.1016/j.jbtep.2015.10.007 26605829

[B9] Carretti B. Borella E. Cornoldi C. De Beni R. (2009). Role of working memory in explaining the performance of individuals with specific reading comprehension difficulties: a meta-analysis. Learn. Individ. Differ. 19, 246–251. 10.1016/j.lindif.2008.10.002

[B10] Clifton C. Ferreira F. Henderson J. M. Inhoff A. W. Liversedge S. P. Reichle E. D. (2016). Eye movements in reading and information processing: Keith Rayner’s 40 year legacy. J. Mem. Lang. 86, 1–19. 10.1016/j.jml.2015.07.004

[B11] Cop U. Drieghe D. Duyck W. (2015). Eye movement patterns in natural reading: a comparison of monolingual and bilingual reading of a novel. PLoS One 10:e0134008. 10.1371/journal.pone.0134008 26287379 PMC4545791

[B13] Deans P. O’Laughlin L. Brubaker B. Gay N. Krug D. (2010). Use of eye movement tracking in the differential diagnosis of attention defecit hyperactivity disorder (ADHD) and reading disability. Psychology 1, 238–246. 10.4236/psych.2010.14032

[B12] de Wit S. J. de Vries F. E. van der Werf Y. D. Cath D. C. Heslenfeld D. J. Veltman E. M.. (2012). Presupplementary motor area hyperactivity during response inhibition: a candidate endophenotype of obsessive-compulsive disorder. Am. J. Psychiatry 169, 1100–1108. 10.1176/appi.ajp.2012.12010073 23032388

[B14] Dittrich W. H. Johansen T. (2013). Cognitive deficits of executive functions and decision-making in obsessive-compulsive disorder. Scand. J. Psychol. 54, 393–400. 10.1111/sjop.12066 23841985

[B15] Dong G. Potenza M. N. (2014). A cognitive-behavioral model of internet gaming disorder: theoretical underpinnings and clinical implications. J. Psychiatr. Res. 58, 7–11. 10.1016/j.jpsychires.2014.07.005 25062755 PMC4448942

[B16] Engbert R. Longtin A. Kliegl R. (2002). A dynamical model of saccade generation in reading based on spatially distributed lexical processing. Vision Res. 42, 621–636. 10.1016/s0042-6989(01)00301-7 11853779

[B17] Feng W. Ramo D. E. Chan S. R. Bourgeois J. A. (2017). Internet gaming disorder: trends in prevalence 1998–2016. Addict. Behav. 75, 17–24. 10.1016/j.addbeh.2017.06.010 28662436 PMC5582011

[B18] Fernández G. Laubrock J. Mandolesi P. Colombo O. Agamennoni O. (2014). Registering eye movements during reading in Alzheimer’s disease: difficulties in predicting upcoming words. J. Clin. Exp. Neuropsychol. 36, 302–316. 10.1080/13803395.2014.892060 24580505

[B19] Fernández G. Mandolesi P. Rotstein N. P. Colombo O. Agamennoni O. Politi L. E. (2013). Eye movement alterations during reading in patients with early Alzheimer disease. Investig. Opthalmology Vis. Sci. 54, 8345–8352. 10.1167/iovs.13-12877 24282223

[B20] Fossati A. Di Ceglie A. Acquarini E. Barratt E. S. (2001). Psychometric properties of an Italian version of the barratt impulsiveness scale-11 (BIS-11) in nonclinical subjects. J. Clin. Psychol. 57, 815–828. 10.1002/jclp.1051 11344467

[B21] García-Madruga J. A. Vila J. O. Gómez-Veiga I. Duque G. Elosúa M. R. (2014). Executive processes, reading comprehension and academic achievement in 3th grade primary students. Learn. Individ. Differ. 35, 41–48. 10.1016/j.lindif.2014.07.013

[B22] Goodman W. K. Price L. H. Rasmussen S. A. Mazure C. Fleischmann R. L. Hill C. L.. (1989). The yale-brown obsessive compulsive scale. I. Development, use and reliability. Arch. Gen. Psychiatry 46, 1006–1011. 10.1001/archpsyc.1989.01810110048007 2684084

[B23] Grant J. E. Potenza M. N. Weinstein A. Gorelick D. A. (2010). Introduction to behavioral addictions. Am. J. Drug Alcohol Abuse 36, 233–241. 10.3109/00952990.2010.491884 20560821 PMC3164585

[B24] Hermans D. Engelen U. Grouwels L. Joos E. Lemmens J. Pieters G. (2008). Cognitive confidence in obsessive-compulsive disorder: distrusting perception, attention and memory. Behav. Res. Ther. 46, 98–113. 10.1016/j.brat.2007.11.001 18076865

[B25] Holden C. (2001). “Behavioral” addictions: do they exist? Science 294, 980–982. 10.1126/science.294.5544.980 11691967

[B26] Itti L. (2015). New eye-tracking techniques may revolutionize mental health screening. Neuron 88, 442–444. 10.1016/j.neuron.2015.10.033 26539886

[B27] Kessler R. C. Angermeyer M. Anthony J. C. De Graaf R. Demyttenaere K. Gasquet I.. (2007). Lifetime prevalence and age-of-onset distributions of mental disorders in the world health organization’s world mental health survey initiative. World Psychiatry 6, 168–176. 18188442 PMC2174588

[B28] Kim S. M. Han D. H. Lee Y. S. Renshaw P. F. (2012). Combined cognitive behavioral therapy and bupropion for the treatment of problematic on-line game play in adolescents with major depressive disorder. Comput. Human Behav. 28, 1954–1959. 10.1016/j.chb.2012.05.015

[B29] Kim S. N. Kim M. Lee T. H. Lee J.-Y. Park S. Park M.. (2018). Increased attentional bias toward visual cues in internet gaming disorder and obsessive-compulsive disorder: an event-related potential study. Front. Psychiatry 9:315. 10.3389/fpsyt.2018.00315 30057559 PMC6053507

[B30] Kim Z. Lee Y. Lee M. (1994). Two-and four-subtest short forms of the Korean-Wechsler adult intelligence scale. Seoul J. Psychiatry 19, 121–126.

[B31] King D. L. Delfabbro P. H. (2014). The cognitive psychology of internet gaming disorder. Clin. Psychol. Rev. 34, 298–308. 10.1016/j.cpr.2014.03.006 24786896

[B32] Metcalf O. Pammer K. (2014). Impulsivity and related neuropsychological features in regular and addictive first person shooter gaming. Cyberpsychol. Behav. Soc. Netw. 17, 147–152. 10.1089/cyber.2013.0024 23971428

[B33] Millan M. J. Agid Y. Brüne M. Bullmore E. T. Carter C. S. Clayton N. S.. (2012). Cognitive dysfunction in psychiatric disorders: characteristics, causes and the quest for improved therapy. Nat. Rev. Drug Discov. 11, 141–168. 10.1038/nrd3628 22293568

[B34] Na E. Choi I. Lee T.-H. Lee H. Rho M. J. Cho H.. (2017). The influence of game genre on internet gaming disorder. J. Behav. Addict. 6, 248–255. 10.1556/2006.6.2017.033 28658960 PMC5520129

[B4] Nilsson Benfatto M. N. Öqvist Seimyr G. Ygge J. Pansell T. Rydberg A. Jacobson C. (2016). Screening for dyslexia using eye tracking during reading. PLoS One 11:e0165508. 10.1371/journal.pone.0165508 27936148 PMC5147795

[B35] Oei A. C. Patterson M. D. (2013). Enhancing cognition with video games: a multiple game training study. PLoS One 8:e58546. 10.1371/journal.pone.0058546 23516504 PMC3596277

[B36] Palmer J. MacLeod C. M. Hunt E. Davidson J. E. (1985). Information processing correlates of reading. J. Mem. Lang. 24, 59–88. 10.1016/0749-596x(85)90016-6

[B37] Park J. H. Han D. H. Kim B.-N. Cheong J. H. Lee Y.-S. (2016). Correlations among social anxiety, self-esteem, impulsivity and game genre in patients with problematic online game playing. Psychiatry Investig. 13, 297–304. 10.4306/pi.2016.13.3.297 27247595 PMC4878963

[B38] Potenza M. N. (2006). Should addictive disorders include non-substance-related conditions? Addiction 101, 142–151. 10.1111/j.1360-0443.2006.01591.x 16930171

[B39] Pretorius E. J. (2002). Reading ability and academic performance in south africa: are we fiddling while rome is burning? Lang. Matters 33, 169–196. 10.1080/10228190208566183

[B40] Raney G. E. Campbell S. J. Bovee J. C. (2014). Using eye movements to evaluate the cognitive processes involved in text comprehension. J. Vis. Exp. 83:e50780. 10.3791/50780 24457916 PMC4089416

[B41] Rayner K. (1998). Eye movements in reading and information processing: 20 years of research. Psychol. Bull. 124, 372–422. 10.1037/0033-2909.124.3.372 9849112

[B42] Rayner K. (2009). Eye movements and attention in reading, scene perception and visual search. Q. J. Exp. Psychol. 62, 1457–1506. 10.1080/17470210902816461 19449261

[B43] Reichle E. D. Rayner K. Pollatsek A. (2003). The E-Z reader model of eye-movement control in reading: comparisons to other models. Behav. Brain Sci. 26, 445–476. 10.1017/s0140525x03000104 15067951

[B44] Robbins T. W. Clark L. (2015). Behavioral addictions. Curr. Opin. Neurobiol. 30, 66–72. 10.1016/j.conb.2014.09.005 25262209

[B45] Sawyer M. G. Whaites L. Rey J. M. Hazell P. L. Graetz B. W. Baghurst P. (2002). Health-related quality of life of children and adolescents with mental disorders. J. Am. Acad. Child Adolesc. Psychiatry 41, 530–537. 10.1097/00004583-200205000-00010 12014785

[B46] Sayyah M. Eslami K. AlaiShehni S. Kouti L. (2016). Cognitive function before and during treatment with selective serotonin reuptake inhibitors in patients with depression or obsessive-compulsive disorder. Psychiatry Res. 2016:5480391. 10.1155/2016/5480391 27597949 PMC5002481

[B48] Shin N. Y. Lee T. Y. Kim E. Kwon J. S. (2014). Cognitive functioning in obsessive-compulsive disorder: a meta-analysis. Psychol. Med. 44, 1121–1130. 10.1017/S0033291713001803 23866289

[B47] Shin M. S. Park S. J. Kim M. S. Lee Y. H. Ha T. H. Kwon J. S. (2004). Deficits of organizational strategy and visual memory in obsessive-compulsive disorder. Neuropsychology 18, 665–672. 10.1037/0894-4105.18.4.665 15506834

[B49] Steenbergen L. Sellaro R. Stock A.-K. Beste C. Colzato L. S. (2015). Action video gaming and cognitive control: playing first person shooter games is associated with improved action cascading but not inhibition. PLoS One 10:e0144364. 10.1371/journal.pone.0144364 26655929 PMC4675555

[B50] Steer R. A. Clark D. A. Beck A. T. Ranieri W. F. (1999). Common and specific dimensions of self-reported anxiety and depression: the BDI-II versus the BDI-IA. Behav. Res. Ther. 37, 183–190. 10.1016/s0005-7967(98)00087-4 9990749

[B51] Steer R. A. Rissmiller D. J. Ranieri W. F. Beck A. T. (1993). Structure of the computer-assisted beck anxiety inventory with psychiatric inpatients. J. Pers. Assess. 60, 532–542. 10.1207/s15327752jpa6003_10 8336268

[B52] Tata P. R. Leibowitz J. A. Prunty M. J. Cameron M. Pickering A. D. (1996). Attentional bias in obsessional compulsive disorder. Behav. Res. Ther. 34, 53–60. 10.1016/0005-7967(95)00041-u 8561765

[B53] Walitza S. Melfsen S. Jans T. Zellmann H. Wewetzer C. Warnke A. (2011). Obsessive-compulsive disorder in children and adolescents. Dtsch. Arztebl. Int. 108, 173–179. 10.3238/arztebl.2011.0173 21475565 PMC3071953

[B54] Young K. S. (1996). Psychology of computer use: XL. addictive use of the internet: a case that breaks the stereotype. Psychol. Rep. 79, 899–902. 10.2466/pr0.1996.79.3.899 8969098

[B55] Zhang Y. Song H. Liu X. Tang D. Chen Y. Zhang X. (2017). Language learning enhanced by massive multiple online role-playing games (MMORPGs) and the underlying behavioral and neural mechanisms. Front. Hum. Neurosci. 11:95. 10.3389/fnhum.2017.00095 28303097 PMC5332359

[B56] Zhou Z. Yuan G. Yao J. (2012). Cognitive biases toward internet game-related pictures and executive deficits in individuals with an internet game addiction. PLoS One 7:e48961. 10.1371/journal.pone.0048961 23155434 PMC3498351

[B57] Zhou Z. Zhou H. Zhu H. (2016). Working memory, executive function and impulsivity in internet-addictive disorders: a comparison with pathological gambling. Acta Neuropsychiatr. 28, 92–100. 10.1017/neu.2015.54 26400106

